# Crave, Like, Eat: Determinants of Food Intake in a Sample of Children and Adolescents with a Wide Range in Body Mass

**DOI:** 10.3389/fpsyg.2016.01389

**Published:** 2016-09-21

**Authors:** Johannes Hofmann, Adrian Meule, Julia Reichenberger, Daniel Weghuber, Elisabeth Ardelt-Gattinger, Jens Blechert

**Affiliations:** ^1^Department of Psychology, University of SalzburgSalzburg, Austria; ^2^Department of Pediatrics, Paracelsus Medical UniversitySalzburg, Austria; ^3^Obesity Research Unit, Paracelsus Medical UniversitySalzburg, Austria; ^4^Obesity Academy AustriaSalzburg, Austria; ^5^Centre for Cognitive Neuroscience, University of SalzburgSalzburg, Austria

**Keywords:** childhood obesity, BMI, food craving, food liking, food intake, food pictures

## Abstract

Obesity is a heterogeneous condition with obese individuals displaying different eating patterns. Growing evidence suggests that there is a subgroup of obese adults that is marked by frequent and intense food cravings and addiction-like consumption of high-calorie foods. Little is known, however, about such a subgroup of obese individuals in childhood and adolescence. In the present study, a sample of children and adolescents with a wide range in body mass was investigated and trait food craving, liking for and intake of high- and low-calorie foods was measured. One-hundred and forty-two children and adolescents (51.4% female, *n* = 73; *M*_age_ = 13.7 years, *SD* = 2.25; *M*_BMI-SDS_ = 1.26, *SD* = 1.50) completed the *Food Cravings Questionnaire-Trait*, then viewed pictures of high- and low-calorie foods and rated their liking for them, and subsequently consumed some of these foods in a bogus taste test. Contrary to expectations, higher body mass was associated with lower consumption of high-calorie foods. However, there was an interaction between body mass and trait food craving when predicting food consumption: in obese participants, higher trait food craving was associated with higher consumption of high-calorie foods and this association was not found in normal-weight participants. The relationship between trait food craving and high-calorie food consumption within obese individuals was mediated by higher liking for high-calorie foods (but not by liking for low-calorie foods). Thus, similar to adults, a subgroup of obese children and adolescents – characterized by high trait food craving – seems to exist, calling for specific targeted treatment strategies.

## Introduction

Obesity remains a global health problem in children, adolescents, and adults (Ng et al., 2014). Contrary to the hopes of young patients with obesity and their families, this disease often carries over into adulthood, alongside several serious and debilitating comorbidities (Whitaker et al., 1997). Adults with obesity, in turn, are likely to pass genetic and environmental vulnerabilities on to their offspring (Moens et al., 2009), which is why there is a need for effective treatments for younger patients to break the cycle. Unfortunately, current lifestyle interventions for obesity have low to moderate long-term success not only in adults (Bischoff et al., 2012), but similarly in adolescents (Moens et al., 2010).

Obesity in childhood and adolescence is determined by interactions between genetic and environmental risk factors, of which parental obesity and parental eating habits appear to be two of the most important ones (Maffeis, 2000; van der Horst et al., 2007). Weight gain results from a positive energy balance and, accordingly, is associated with low physical activity (Maffeis, 2000). However, findings about excessive energy intake in obese individuals are inconsistent: whereas some epidemiological studies do find an association between energy intake and body mass (Vandevijvere et al., 2015), others do not (Heini and Weinsier, 1997; Maffeis, 2000). A recent study, for example, even showed that, when combined with low energy expenditure, *low* energy intake predicted weight gain (Hume et al., 2016).

Research in this area is further complicated by the documented underreporting of caloric intake, particularly in those with obesity (Platte et al., 1995; Kretsch et al., 1999; Stice et al., 2015). Furthermore, food environments of obese individuals differ to those of non-obese individuals due to different socioeconomic conditions, leading to an overexposure to low-quality, energy-dense and processed foods. This represents a critical confound when it comes to an investigation of high- versus low-calorie food choices. Laboratory studies account for that confound by presenting comparable food options to all participants regardless of body weight (or socioeconomic status). Under such conditions, however, findings on overconsumption are also inconclusive with some studies showing higher food intake in obese compared to normal-weight adults (e.g., Laessle et al., 2007) or similar food intake in obese and normal-weight adults (e.g., Shah et al., 2014).

It has been described early on that obesity represents a heterogeneous condition and different eating patterns in obese individuals can be found (Stunkard, 1959). Accordingly, researchers have identified subgroups within obese samples by means of different eating styles. In adults, for example, obese individuals with binge eating have been compared with obese individuals without binge eating (e.g., Schulz and Laessle, 2012; Dalton and Finlayson, 2014) whereas studies in children and adolescents have focused on individuals with and without loss of control eating (e.g., Tanofsky-Kraff et al., 2009; Hartmann et al., 2010). In recent years, an increasing number of studies has investigated obese adolescents and adults with and without addiction-like eating behavior (Davis et al., 2011, 2013; Meule et al., 2014b; Burrows and Meule, 2015; Meule et al., 2015). Importantly, there is a strong overlap between all these concepts (e.g., Schulte et al., 2016). Accordingly, correlates of these obese subtypes are largely similar, irrespective of whether binge eating, loss of control eating, or addiction-like eating is used to define them. For example, Dalton and Finlayson (2014) found that obese adults with binge eating experienced more frequent and more intense food cravings and showed higher implicit liking for and consumed more high-fat sweet foods than obese adults without binge eating. Similarly, children and adolescents with loss of control eating were more impulsive and consumed more high-calorie snack and dessert-type foods in the laboratory than those without loss of control eating (Tanofsky-Kraff et al., 2009; Hartmann et al., 2010). Finally, obese adolescents and adults with an addiction-like eating behavior were found to be more impulsive and to experience more frequent food cravings than obese adolescents and adults without this addiction-like eating behavior (Davis et al., 2011, 2013; Meule et al., 2014b, 2015). To conclude, it appears that there is a subgroup of obese individuals (including both children, adolescents, and adults), which is marked by high impulsivity, high preference for high-calorie foods, and frequent and intense experiences of food cravings, which result in excessive food consumption (which may be conceptualized as loss of control eating, binge eating, or addiction-like eating).

What this overview illustrates is that several different concepts have been used to describe different subtypes within obese samples based on their eating style (e.g., loss of control eating, binge eating, or addiction-like eating). Yet, we would argue that one core theme behind all these concepts is the experience of frequent and intense food cravings, as indicated above. Food craving refers to an intense desire to consume a specific type of food and, accordingly, is often associated with consumption of that food (Martin et al., 2008). While experiencing food craving momentarily is a transient state, frequent experiences of food cravings can also be considered as a trait (Cepeda-Benito et al., 2000). For example, the Food Cravings Questionnaire-Trait (FCQ-T) measures cognitive, affective, and behavioral aspects of food craving experiences, with higher scores indicating more frequent food cravings (i.e., higher “trait food craving”; Cepeda-Benito et al., 2000). The conceptualization of food craving as a trait has been supported by high stability of FCQ-T scores over 6 months (Meule et al., 2014a). Moreover, validity of the concept has been supported by findings showing that adults with high trait food craving scores are more susceptible for experiencing food-cue elicited craving in the laboratory (e.g., Meule et al., 2012b, 2014c), have an automatic approach bias toward high-calorie food cues (Brockmeyer et al., 2015a), and show reward-related brain activations in response to high-calorie food cues (Ulrich et al., 2016). Finally, higher FCQ-T scores are strongly associated with loss of control eating frequency, binge eating severity, and addiction-like eating in adolescents and adults (e.g., Meule and Kübler, 2012; Meule et al., 2012a, 2015; Davis et al., 2013; Innamorati et al., 2015).

To date, however, no study has investigated liking for and consumption of foods as a function of trait food craving and body mass in children and adolescents. Based on the above-mentioned findings, it was expected that body mass would be positively correlated with the energy density of foods consumed in the laboratory. In other words, obese children and adolescents were expected to show a higher tendency to consume high-calorie foods than normal-weight children and adolescents (hypothesis 1). This effect was expected to interact with trait food craving: higher trait food craving was expected to relate to a higher tendency to consume high-calorie foods, particularly in obese participants (hypothesis 2). That is, obese participants with high trait food craving scores were expected to eat the most energy dense foods. Finally, as an exploratory goal, possible mediators of such an effect were tested. Specifically, preferential selection of high-calorie foods in obese children and adolescents with high trait food craving could be mediated by higher liking for these foods, but also by lower liking for low-calorie foods (hypothesis 3).

## Materials and Methods

### Participants

The study was approved by the ethical review board of the University of Salzburg and all participants (and, when appropriate, their parents) signed informed consent. A total of 161 participants (without food allergies) were recruited through the obesity center at the Paracelsus Medical University and from public schools in Salzburg, Austria. Nineteen participants had to be excluded due to missing data. For the remaining 142 participants (73 female, 51.4%), age ranged between 10–18 years (*M* = 13.7, *SD* = 2.25). Body mass index standard deviation score (BMI-SDS) ranged between -2.20 and 3.60 (*M* = 1.26, *SD* = 1.50), based on German reference values (Kromeyer-Hauschild et al., 2001). According to cut-offs based on the recommendations by the European Childhood Obesity Group (Rolland-Cachera, 2011), three participants (2.11%) were underweight (BMI-SDS < -2.00), 56 participants (39.4%) were normal-weight (-2.00 < BMI-SDS < 1.00), 19 participants (13.4%) were overweight (1.00 < BMI-SDS < 2.00) and 64 participants (45.1%) were obese (BMI-SDS > 2.00).

### Food Cravings Questionnaire-Trait (FCQ-T)

Trait food craving was assessed with the German version of 39-item FCQ-T (Cepeda-Benito et al., 2000; Meule et al., 2012a). Items (e.g., “If I give in to a food craving, all control is lost.,” “If I am craving something, thoughts of eating it consume me.”) are scored on a six-point scale with response categories ranging from *never/not applicable* to *always*. The scale contains several subscales. However, factor structure could not be replicated in several studies (cf. Rodríguez-Martín and Meule, 2015). Moreover, internal consistency of the scale is usually very high and, accordingly, subscale scores are highly correlated with each other (ibid.). Therefore, only the total score was used and internal consistency was Cronbach’s α = 0.976 in the current study.

### Procedure

Participants were instructed to abstain from eating for at least 3 h prior to testing to ensure that participants were hungry and, thus, to create a typical mealtime condition during testing. Participants were tested individually and completed the FCQ-T among other questionnaires in the laboratory. The study also included EEG recording amongst other measures, results of which are described elsewhere (Hofmann et al., 2015). Participants passively viewed pictures of food on a monitor. Stimuli comprised 32 images of food with low energy density (e.g., apple, kiwi, broccoli, tomato) and 32 images of food with high energy density (e.g., chocolate, peanuts, cookies, cheese), which were selected from *food-pics*, a database of standardized food and non-food images with high familiarity and recognizability (Blechert et al., 2014)^1^. Mean energy density of the low-calorie foods was *M* = 60.6 kcal/100 g (*SD* = 89.4) and mean energy density of the high-calorie foods was *M* = 449 kcal/100 g (*SD* = 99.1). Mean calories displayed on the images were *M* = 114 kcal/image (*SD* = 117) for the low-calorie foods and *M* = 275 kcal/image (*SD* = 224) for the high-calorie foods. Images were presented in pseudorandom sequence for 2 s each, interspersed by a variable fixation intertrial-interval (1000 ± 200 ms). Each image was repeated once, totaling in 128 image presentations. Participants rated their liking for each food on-screen on a visual analog scale (“How palatable do you consider the displayed food?”), ranging from 0 (not at all) to 100 (very much). After this picture viewing task, participants were handed a sheet with a subset of the food images displayed before (16 low-calorie and 16 high-calorie foods) and were instructed to select seven of them for a following taste test. Participants were served the selected foods and instructed to taste from each food. They were also told that they could eat as much as they wanted. Then, the experimenter left the room until participants indicated that they were finished. Finally, body weight and height was measured and the remaining foods were weighed.

### Data Analyses

On average, participants consumed *M* = 3.88 (*SD* = 1.63) high-calorie foods, indicating that participants selected both low- and high-calorie foods and ruling out the possibility they did not like the low-calorie foods^2^. As food choice was limited to a fixed number, selection of either low-calorie or high-calorie foods speaks to a relative preference (i.e., low-calorie foods cannot be analyzed separately or independently from high-calorie foods). Thus, to arrive at a continuous index of relative preference for energy dense foods, all selected foods were combined and their average energy density computed (in kcal/100 g). Thus, higher values indicate a preference to select and consume high-calorie foods. Liking ratings were averaged for high-calorie and low-calorie foods separately to allow a test of our exploratory mediation analysis.

To test *hypothesis 1*, correlations between study variables were computed. Here, a positive correlation between BMI-SDS and mean energy density of consumed foods would indicate a relative preference for energy-dense foods in those with higher body mass. To test *hypothesis 2*, a linear regression analysis was calculated with BMI-SDS, FCQ-T scores, and their interaction as predictors of mean energy density of consumed foods. Predictor variables were mean-centered before calculating the product term in order to ease interpretation of the single predictors (Hayes, 2013). A significant interaction was followed up by examining the association between trait food craving and mean energy density of consumed foods at low (-1 *SD*) and high (+1 *SD*) values of BMI-SDS (Aiken and West, 1991). Note that, given the mean and standard deviation of the current sample (see Participants section), these values corresponded to normal-weight participants and obese participants, respectively.

**FIGURE 1:**
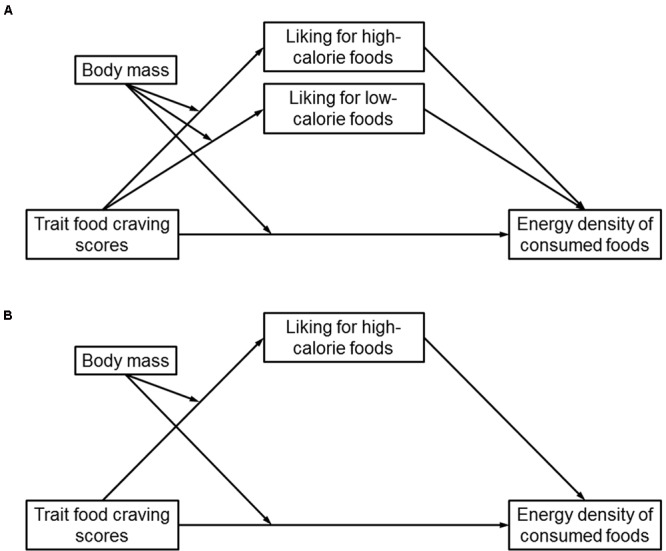
**(A)** Conceptual moderated mediation model, in which trait food craving scores, body mass, and their interaction were used as predictors of liking for high- and low-calorie foods (as parallel mediators) and mean energy density of consumed foods. **(B)** Empirical moderated mediation model, in which body mass moderated the indirect effect of trait food craving scores on mean energy density of consumed foods via liking for high-calorie foods. Liking for low-calorie foods did not mediate the interactive effect of trait food craving scores and body mass on mean energy density of consumed foods.

To explore mediation effects of liking for high- and low-calorie foods in the relationship of body mass and trait food craving with mean energy density of consumed foods (*hypothesis 3*), a moderated mediation model was tested with PROCESS for SPSS (Hayes, 2013). Specifically, model no. eight in PROCESS was chosen with trait food craving as independent variable, liking for high- and low-calorie foods as parallel mediators, mean energy density of consumed foods as outcome variable, and body mass as moderator (**Figure 1A**). Practically, this means that the above-mentioned moderation model, which tested the interactive effect between body mass and trait food craving on mean energy density of consumed foods, was extended by additionally testing the interactive effect between body mass and trait food craving when predicting liking for high- and low-calorie foods and, thus, this model enables to test an indirect effect of body mass × trait food craving on mean energy density of consumed foods via liking for food. Indirect (i.e., mediating) effects were evaluated with 95% bias-corrected confidence intervals based on 10,000 bootstrap samples. When the confidence interval does not contain zero, this means that the indirect effect can be considered statistically significant (Hayes, 2013). If the presence of such an indirect effect depends on the value of a moderating variable (here: BMI-SDS), this is an indication of moderated mediation.

## Results

### Correlations between Study Variables (Hypothesis 1)

Contrary to hypothesis 1, BMI-SDS was negatively correlated with mean energy density of consumed foods (**Table 1**). Body mass also correlated negatively with liking for high-calorie foods. Trait craving, by contrast, correlated positively with mean energy density of consumed foods and with liking for high-calorie foods. Liking for high-calorie foods correlated positively and liking for low-calorie foods correlated negatively with mean energy density of consumed foods (**Table 1**).

**Table 1:** Descriptive statistics of and correlations between study variables.

*N* = 142	*M*	*SD*	Range	1	2	3	4	5
1. Standardized body mass index	1.26	1.50	-2.20–3.60	–	0.001	**-0.220**	0.152	**-0.227**
2. Food Cravings Questionnaire-Trait (FCQ-T)	84.4	37.0	39.0–219		–	**0.277**	-0.100	**0.233**
3. Liking for high-calorie foods	64.1	18.3	4.37–94.5			–	**0.296**	**0.370**
4. Liking for low-calorie foods	64.9	16.9	2.22–98.1				–	**-0.318**
5. Energy density of consumed foods (kcal/100 g)	289	95.1	66.7–523					–

*Bold formatting indicates *p* < 0.05.*

### Moderation Analysis (Hypothesis 2)

**FIGURE 2:**
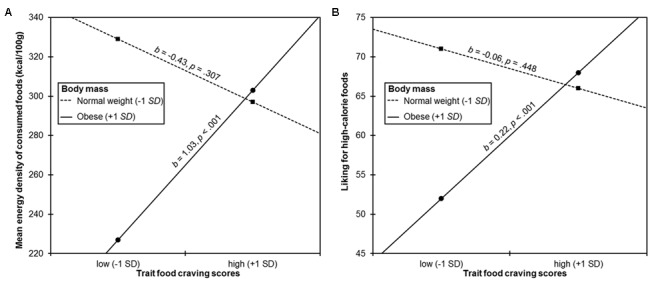
**Simple slopes probing the interaction between trait food craving scores and body mass when predicting **(A)** mean energy density of consumed foods and **(B)** liking for high-calorie foods.** Trait food craving scores positively predicted mean energy density of consumed foods and liking for high-calorie foods in obese participants (+1 *SD* of mean standardized body mass index), but not in normal-weight participants (-1 *SD* of mean standardized body mass index).

The interaction between body mass and trait food craving scores when predicting mean energy density of consumed foods was significant (**Table 2**). Partially in line with hypothesis 2, trait food craving scores positively predicted mean energy density of consumed foods in obese participants, but not in normal-weight participants (**Figure 2A**). However, obese participants with high levels of trait food craving did not show the highest preference for high-calorie foods.

**Table 2:** Results from linear regression analyses with trait food craving scores and body mass predicting liking for high- and low-calorie foods and mean energy density of consumed foods.

	Liking for high-calorie foods			Liking for low-calorie foods			Mean energy density of consumed foods		
	*b*	*SE*	*p*	*b*	*SE*	*p*	*b*	*SE*	*p*
Food Cravings Questionnaire-Trait (FCQ-T)	-0.04	0.08	0.596	-0.07	0.08	0.364	-0.32	0.39	0.411
Standardized body mass index (BMI-SDS)	**-3.04**	**0.95**	**0.002**	1.68	0.95	0.081	**-16.3**	**5.02**	**0.002**
FCQ-T × BMI-SDS	**0.09**	**0.03**	**0.007**	0.01	0.03	0.731	**0.49**	**0.18**	**0.007**

*Bold formatting indicates *p* < 0.05.*

### Moderated Mediation Analysis (Hypothesis 3)

The interaction between body mass and trait food craving scores was significant when predicting liking for high-calorie foods, but not when predicting liking for low-calorie foods (**Table 2**). Trait food craving scores positively predicted liking for high-calorie foods in obese participants, but not in normal-weight participants (**Figure 2B**). In partial agreement with hypothesis 3, there was an indirect effect of trait food craving scores on mean energy density of consumed foods via liking for high-calorie foods in obese participants (bootstrap estimate 0.50, 95% CI [0.22, 0.86]), but not in normal-weight participants (bootstrap estimate -0.14, 95% CI [-0.53, 0.25]). There was no mediation effect of liking for low-calorie foods (bootstrap estimate 0.09, 95% CI [-0.22, 0.43], for obese participants; bootstrap estimate 0.17, 95% CI [-0.33, 0.76], for normal-weight participants). Including age as covariate in the current analyses did not change interpretation of results.

The empirical moderated mediation model is displayed in **Figure 1B** and can be summarized as follows: body mass and trait food craving interactively predicted mean energy density of consumed foods such that higher trait food craving was associated with preferential selection of high-calorie foods, but only in obese participants. Examining indirect effects revealed that the interactive effect between body mass and trait food craving on mean energy density of consumed foods was mediated by liking for high-calorie foods. That is, higher trait food craving was associated with higher liking for high-calorie foods in obese individuals, which was in turn related to preferential selection of high-calorie foods. Although higher liking for low-calorie foods was indeed related to lower mean energy density of consumed foods (**Table 1**), liking for low-calorie foods did not mediate the interactive effect of body mass and trait food craving on mean energy density of consumed foods (**Figure 1B**).

## Discussion

A first aim of this study was to investigate food choice and consumption in children and adolescents as a function of body mass in the laboratory. It was expected that a higher body mass would relate to a higher tendency to select and consume high-calorie foods (hypothesis 1). Contrary to expectations, however, the opposite was found: higher body mass was associated with a tendency to select foods with a lower energy density. In addition, higher body mass was related to a lower liking for high-calorie foods. It may be speculated that these results are due to demand characteristics in laboratory settings and impression management displayed by overweight and obese participants. For example, it has been found that participants show lower laboratory food intake when they expect that food intake is measured than when they are unaware of food intake measurement (Robinson et al., 2015). Furthermore, while it has been found that obese children eat more calories and choose more unhealthy snacks than normal-weight children in the laboratory when alone, this effect cannot be found when they are accompanied by others (Salvy et al., 2007, 2008). In addition, overweight children consumed more healthy snacks than normal-weight children in one of these studies (Salvy et al., 2008) and reported lower appetite than normal-weight children in another study (Jansen et al., 2003). As participants in the current study knew that they were observed by the experimenter during the taste test, it is likely that overweight participants reduced their selection of high-calorie foods due to these social effects.

Hypothesis 2 predicted interactive effects between body mass and trait food craving when predicting food choice and consumption. It was expected that higher body mass would be particularly related to a higher tendency to select and consume high-calorie foods when trait food craving was also high. While the presence of an interactive effect between body mass and trait food craving was confirmed, it could not been shown that obese participants with high levels of trait food craving had the highest preference for high-calorie foods. Instead, it appeared that trait food craving compensated the overall negative association between body mass and mean energy density of consumed foods. While obese participants showed a lower preference for high-calorie foods than normal-weight participants did in general, obese participants with high trait food craving showed a similar preference for high-calorie foods like normal-weight participants (**Figure 2A**). Thus, it appears that while some obese participants successfully managed to avoid high-calorie foods in the present study, those with high trait food craving did not achieve this, which may be due to higher reward sensitivity and impulsivity as compared to obese individuals with low trait food craving. Hence, results are in line with the subtyping approaches described above (e.g., Dalton and Finlayson, 2014), suggesting that there is a subset of individuals with a high preference and frequent cravings for high-calorie foods within the population of obese children and adolescents. Interestingly, trait food craving scores were associated with selection of foods only in obese participants, but not in normal-weight participants, although trait food craving scores were uncorrelated with body weight. Thus, it appears that although there also were normal-weight children and adolescents with high trait food craving scores, they did not show this preferential selection of high-calorie foods in the current study and this behavior may have prevented them from becoming obese in the first place. Future studies are necessary, which elucidate the mechanisms that enable normal-weight individuals with high trait food craving to refrain from giving in to their cravings and, as a result, to stay lean.

A third aim of the current study was to explore mediating effects that may explain associations between body mass, trait food craving, and mean energy density of consumed foods. Partially in line with hypothesis 3, it was found that the positive association between trait food craving and preferential selection of high-calorie foods in obese individuals was mediated by higher liking for these foods. While the temporal order of measuring these variables corresponded to the order of the statistical mediation model (trait food craving → food liking → food selection), causal directions must be interpreted with caution. Specifically, while being a high trait food craver may increase the likelihood of preferring high-calorie foods, it may as well be that food preferences that develop early in life (i.e., liking for high-calorie foods) may increase the likelihood of becoming a high trait food craver in later childhood and adolescence.

Theoretically, it would have been plausible that obese individuals with high trait food craving may select more high-calorie foods merely because they do not like low-calorie foods. This possibility, however, was ruled out in the current study. Obese individuals with high trait food craving indicated to like low-calorie foods just as much as obese individuals with low trait food craving and a higher tendency to select high-calorie foods was specifically related to higher liking for these foods. These results are line with the findings by Dalton and Finlayson (2014), which showed that obese adults with binge eating did not differ from obese adults without binge eating in their intake of low-calorie foods, but that obese adults with binge eating selectively showed higher intake of high-fat sweet foods. Therefore, we would expect that the mechanisms found in the current study (high trait food craving → liking for high-calorie foods → consumption of high-calorie foods) may similarly apply to related samples such as children and adolescents with loss of control eating, binge eating, or addiction-like eating (Tanofsky-Kraff et al., 2009; Meule et al., 2015).

Several aspects limit interpretation of the current results. First, alternative explanations (e.g., for the reduced selection and consumption of high-calorie foods in obese participants) cannot be fully excluded. For example, results may have been influenced by the recruitment procedure in the current study. Specifically, most obese participants were recruited from the obesity center of the local hospital, where some underwent lifestyle interventions targeting unhealthy eating styles after the laboratory assessment. As a result, they might have monitored their eating more closely than individuals with lower weight. Another possibility refers to familiarity with the foods presented. Although only foods with a high familiarity and recognizability in adults were selected, familiarity was not assessed in the current study and, thus, may have influenced food choice in our sample of children and adolescents. Second, the current study investigated a sample with a large age range and it has been previously reported that adolescents have elevated reward sensitivity as compared to both children and adults (Galván, 2013). Although controlling for age in the current analyses did not change results, future studies with a larger number of participants in each age group are necessary to determine if similar differences between children and adolescents can be found when examining the interrelations between body weight, trait food craving, food liking, and food choice. Third, while the FCQ-T has been extensively employed in adult samples, it has not been validated in children and adolescents yet. However, internal consistency in the current study was high and of similar magnitude as has been found in studies with adults (Meule et al., 2012a) and in a study with adolescents (Meule et al., 2015), which supports its feasibility in lower age groups.

Consistent with conceptualizations in obese adults (e.g., trait binge eating or addiction-like eating subtypes; Davis et al., 2013; Dalton and Finlayson, 2014) and with findings in children and adolescents (Tanofsky-Kraff et al., 2009), the present results support that a subset of obese children and adolescents show a higher preference and more frequent cravings for high-calorie foods than other obese children and adolescents. However, future studies may also address the question how food intake and development of obesity can be explained in obese children and adolescents with low trait food craving. For example, it has been found that although children with loss of control eating differed from those without loss of control eating in food choice, no differences in total energy intake was observed (Tanofsky-Kraff et al., 2009). Likewise, obese adults with binge eating disorder exhibited a faster eating rate and ingested larger spoonfuls than those without binge eating disorder in the laboratory, but did not differ in the total amount of energy consumed (Schulz and Laessle, 2012). Thus, it appears that even the subgroup of obese individuals without loss of control or binge eating consumes large amounts of energy, mechanisms of which need to be identified in future studies.

Given these findings, future obesity treatments should acknowledge differences within the population of obese children and adolescents and tailor treatment strategies according to individual eating styles instead of assuming homogeneity (Green et al., 2016). In obese adults, treatment protocols that differentiate between those with or without binge eating show higher success rates than when obese patients are treated as a homogenous group (Grilo et al., 2011). Compared to untailored interventions, individualized approaches have already been shown to have better long-term effects in childhood obesity treatment as well (Taylor et al., 2015). Recent advancements in obesity treatment focus on temptation management by use of different strategies such as temptation resistance and temptation prevention (Appelhans et al., 2016) or include behavioral trainings to automatize avoidance responses or devaluate palatable food cues (Brockmeyer et al., 2015b; Jones et al., 2016). While these approaches represent promising tools for obesity treatment, they may be particularly suited for some obese individuals (e.g., those with frequent food cravings and eating binges), but may be ineffective in others (e.g., those with rather modest average daily excess of energy intake over energy expenditure in the absence of frequent craving episodes and eating binges). The current results also highlight the need for early obesity prevention efforts. As food preferences are formed early in life (Ventura and Worobey, 2013), early shaping of preferences for healthy foods could help to reduce liking and craving for unhealthy foods.

## Conclusion

The present results suggest that obese children and adolescents do not generally overconsume or display elevated liking for high-calorie foods. Instead, there seems to be a subgroup within the group of obese children and adolescents, which is characterized by frequent experiences of food craving and displays a higher preference for high-calorie foods than other obese individuals. This differentiation as a function of trait food craving was specific for obese individuals as it could not be found for normal-weight individuals. Finally, this differentiation was specific in that it was mediated by higher liking for high-calorie foods (but not lower liking for low-calorie foods), suggesting a possible mechanism that can account for why obese children and adolescents with high trait food craving preferentially consume high-calorie foods compared to those with low trait food craving.

## Author Contributions

Design, recruitment, implementation, analysis, and writing: JH and JB. Analysis and writing: AM and JR. Design, recruitment, and writing: DW and EA.

## Conflict of Interest Statement

The authors declare that the research was conducted in the absence of any commercial or financial relationships that could be construed as a potential conflict of interest.

## Footnotes

1 Picture numbers in the food-pics database: 4, 8, 18, 26, 62, 63, 70, 104, 110, 111, 117, 147, 148, 149, 152, 153, 154, 155, 159, 168, 169, 170,171, 173, 175, 176, 177, 180, 183, 185, 192, 193, 194, 197, 198, 200, 202, 206, 208, 210, 224, 227, 237, 241, 244, 249, 250, 251, 252, 254, 255, 256, 265, 267, 268, 271, 272, 273, 281, 282, 285, 286, 287, 303.

2 Note that participants were instructed to taste from each food that they had selected and, therefore, the number of selected high- and low-calorie foods equals the number of consumed high- and low-calorie foods. Similarly, the total number of calories selected was highly correlated with the total number of calories consumed (*r* = 0.702, *p* < 0.001).
